# Genetic diversity of the *Plasmodium falciparum msp*2 gene in children from the Eastern Democratic Republic of Congo: a comparative study of clinical and subclinical infections

**DOI:** 10.1186/s12920-026-02413-7

**Published:** 2026-06-13

**Authors:** Moski Morisho Nasibu, Lambert Morisho Mulakwa, Serge Muganda Chirimwami, Charles Bamavu Amisi, Justin Byamungu Ahadi, Rodrigue K. Zakitoka, André N. H. Bulabula, Ali Bulabula

**Affiliations:** 1 Department of Microbiology-Parasitology, Faculty of Medicine, University of Kindu, Boulevard Joseph Kabila Number 006, Kindu, Democratic Republic of the Congo; 2 Center for Tropical Diseases and Global Health, Faculty of Medicine, Catholic University of Bukavu, Bukavu, Democratic Republic of the Congo; 3 Department of General Care, Kanyamulande Higher School of Medical Techniques, P.O. Box: 1247, Governor Street Number 006, Bukavu, Democratic Republic of the Congo; 4 Department of General Care, Kasongo Higher School of Medical Techniques, Kindu, Democratic Republic of the Congo; 5 Preventive and Curative Service, Biomedical Research Unit, Vaccine and Antimicrobial Resistance, Muhungu-Etat/CELPA Health Center, P.O. Box: 1247, Bukavu, Democratic Republic of the Congo; 6 Vaccination Service, French Red Cross Health and Prevention Center in Kourou, Kourou, French Guyana France

**Keywords:** *Plasmodium falciparum*, Genetic diversity, Asymptomatic infection, *Merozoite surface protein* 2 (*msp2*), Democratic Republic of Congo

## Abstract

**Background:**

*Plasmodium falciparum* is the main cause of malaria-related illness and death in sub-Saharan Africa. Despite control efforts, asymptomatic carriers, particularly children, continue to sustain transmission. Improving surveillance requires a better understanding of the genetic diversity and parasite burden of symptomatic versus asymptomatic infections.

**Objective:**

To compare *Plasmodium falciparum* genotypic profiles and *msp*2 gene copy numbers in symptomatic and asymptomatic children in Kindu, and assess associations with clinical status.

**Methods:**

Children aged 6 months to 12 years were recruited from health centres and schools. DNA extracted from dried blood spots (Chelex^®^ 100) was analysed by nested PCR to identify *msp*2 allelic families (FC27, 3D7, multiplicity of infection) and by quantitative PCR to estimate copy number. Statistical tests compared groups and predictors of symptomatic infection.

**Results:**

Of the 522 children included, those in the symptomatic group were younger than those in the asymptomatic group (mean age: 5.77 vs. 7.25 years; *p* < 0.001) and were more frequently male (70%). *P. falciparum* prevalence was higher among symptomatic children (69.8%). The FC27 allele family and polyclonal infections (higher multiplicity of infection) were strongly associated with symptomatic malaria (aOR = 6.50 and 24.58, respectively; *p* < 0.01). Gene copy number was higher in symptomatic and polyclonal infections, but was not independently associated with clinical status after multivariable adjustment (aOR = 0.98; *p* = 0.134). Parasite positivity remained a strong independent predictor of symptomatic disease (aOR = 3.83; *p* < 0.001).

**Conclusion:**

Symptomatic malaria in children is more closely associated with parasite genotype diversity than density. This emphasises the importance of control strategies for molecular surveillance.

## Introduction

Malaria remains one of the most significant global health threats, with an estimated 263 million cases and approximately 597,000 deaths reported worldwide in 2023, according to the World Health Organization (WHO) in 2024. Sub-Saharan Africa (SSA) continues to bear the heaviest burden, accounting for 94% of all cases and 95% of malaria-related deaths, of which 76% occurred in children under the age of five. The persistent high mortality rate is attributed to multiple converging factors, including increasing resistance to antimalarial drugs and insecticides, the effects of climate change, humanitarian crises, and chronic underfunding of control programs [1–5]. *Plasmodium falciparum (P. falciparum)*, the most virulent species, is responsible for the vast majority of severe cases and deaths. Although current malaria control strategies prioritise treating symptomatic infections, asymptomatic carriers individuals who are infected but show no clinical signs are increasingly recognised as a substantial and often undetected reservoir that sustains transmission within communities [6–8].

Understanding the genetic diversity of *P. falciparum* is critical to improving both surveillance and targeted interventions. Molecular markers such as the *merozoite surface protein* 2 (*msp*2) gene provide valuable insights into parasite population structure. The *msp*2 gene is highly polymorphic and encodes a protein involved in the asexual blood stage, with two major allelic families (FC27 and 3D7) frequently used for genotyping and assessing the multiplicity of infection [9, 10]. Quantifying *msp2* copy number using real-time quantitative PCR (qPCR) also allows for a more sensitive estimation of parasite burden, particularly in submicroscopic or low-density infections [11, 12].

Although several studies have investigated the allelic diversity of *P. falciparum* in Democratic Republic of the Congo (DRC), few have directly compared the genetic profiles of isolates from symptomatic and asymptomatic children, especially in urban settings such as Kindu town. Moreover, the relationship between specific *msp*2 alleles and clinical outcomes remains unclear, with inconsistent evidence regarding their association with disease severity or asymptomatic carriage.

This study aims to investigate the allelic diversity and parasite burden of *P. falciparum* in symptomatic and asymptomatic children aged 6 months to 12 years in Kindu. Using a combination of nested PCR and qPCR targeting the *msp*2 gene, we sought to explore potential associations between genetic profiles and clinical presentation, thereby contributing to a better understanding of transmission dynamics and informing future surveillance strategies.

## Material and method

### Study design and setting

This cross-sectional observational study with descriptive and analytical objectives was carried out using a multidisciplinary approach combining epidemiology and molecular biology. The research was conducted by the parasitology department at the University of Kindu laboratory and its partner network between July and December 2024. The biological samples analysed were collected in Kindu, a city in the Maniema province of the DRC, between October 2023 and July 2024. They were obtained from Kindu and Alunguli general referral hospitals, and the surrounding urban areas of Basoko, Braza and Route Kalima.

### Study population

Children aged between six months and twelve years were enrolled in the study. Symptomatic participants were identified based on the presence of a fever and recruited during outpatient consultations or hospital admissions of both referral hospitals. Asymptomatic children, defined as those without a fever, recruited during hospital visits for non-febrile conditions and through routine pre-school consultations at local health centres, as well as during school-based visits in the target surrounding urban areas.

### Inclusion criteria

To be included in the symptomatic group, children had to have a body temperature of at least 38.0 °C and either a reported history of fever within the previous 24 h or a positive rapid diagnostic test (RDT). They also had to exhibit at least one malaria-related clinical feature, as defined by WHO guidelines. Children in the asymptomatic group were eligible if they had no fever or malaria-related symptoms within seven days of sample collection. Written informed consent was obtained from the parent or legal guardian of each participant.

### Non-inclusion criteria

Children whose parent or legal guardian did not provide informed consent were excluded from the study.

### Biological analyses

#### Sample collection

Peripheral blood samples were collected from each participant either by venipuncture into EDTA-coated tubes or, in younger children, by finger prick with sterile disposable lancets, where malaria RDT or thick blood smears were performed to identify *Plasmodium*. Upon collection, samples were immediately centrifuged at 1500 rpm for 10 min to separate plasma and cellular components. Red blood cells (RBC), pellets were then carefully aliquoted and stored at -20 °C until genomic DNA extraction for downstream molecular analyses.

#### Malaria diagnosis

Malaria diagnosis involved both microscopy and rapid diagnostic testing. Thick blood smears were examined using the Lambaréné method to quantify parasite density. Thin blood smears were prepared and stained with Giemsa for microscopic examination in order to identify the *Plasmodium* species. The OptiMAL-IT^®^ rapid diagnostic test (RDT) was then performed on the samples to detect *Plasmodium* lactate dehydrogenase (*pLDH*), including the *P. falciparum*-specific isoform (*pfLDH*).

### Molecular analysis

Genomic DNA was extracted from red blood cells using the EZNA^®^ blood DNA kit, following the manufacturer’s protocol. The procedure involved red cell lysis, protein digestion, ethanol precipitation, and column purification. DNA was eluted in 90 µL of pre-warmed nuclease-free water and stored at -20 °C until analysis.

#### Nested PCR for *Plasmodium* species identification and genotyping

For *Plasmodium* species detection and genotyping, nested polymerase chain reaction (PCR) was performed in sequential steps. The first round of PCR targeted the 18 S rRNA gene to detect *Plasmodium* spp., followed by a second amplification using species-specific primers for *P. falciparum*. Genotyping of *P. falciparum* strains was further performed by amplifying the polymorphic *merozoite surface protein* 2 (*msp*2) gene, allowing identification of the FC27 and 3D7 allelic families. Each 25 µL reaction mixture contained 0.024 U of Taq DNA polymerase, 1× reaction buffer, 1.5 mM MgCl₂, 0.2 mM dNTPs, and 0.8 µM of each forward and reverse primer. For the primary reaction (PCR1), 10 µL of extracted genomic DNA was used as the template. For the nested reaction (PCR2), 2 µL of PCR1 product was used. Amplification was carried out in a Bio-Rad^®^ thermocycler. The thermal cycling conditions were as follows: initial denaturation at 95 °C for 5 min, followed by 30 cycles of denaturation at 94 °C for 1 min, annealing at 60 °C for 2 min, and extension at 72 °C for 2 min. PCR products were separated by electrophoresis on 2% agarose gels and visualized under ultraviolet light at 520 nm using a Vilber E-Box imaging system (Table 1).

**Table 1:** Sequences of primers used for molecular diagnosis and expected band sizes

Species	Primers	Primer sequences (5’-3’)	Expected sizes (bp)
*Plasmodium spp.*	rPLU5	CCTGTTGTTGCCTTAAACTTC	1100
	rPLU6	TTAAAATTGTTGCAGTTAAAACG	
*P. falciparum*	rFAL1	TTAAACTGGTTTGGGAAAACC AAATATATT	205
	rFAL2	ACACAATGAACTCAATCATGA CTACCCGTC	
*Pfmsp2*	PfMSP2-FC	ATGAAGGTAATTAAAACATTGTTCTCTAA	200–600
	PfMSP2-RC	CTTTGTTCATACTGCAATACATAAACA	
FC27	FC27-F	GCATTGCCAGAACTTGAA	250–400
	FC27-R	GGAGGGATCTTCGTAAGGA	
3D7/IC	3D7-F	AGTATCAAGAAGAGGAACTGG	250–400
	3D7-R	GAGGGATGTTGCTGCTCCACAG	

*Plamodium spp.* (*Plasmodium* species), *Pfmsp2* (*Plasmodium falciparum* merozoite surface protein 2), *P. faciparum* (*Plasmodium falciparum*)

#### Quantification of *Plasmodium falciparum* parasite burden by qPCR

To assess *P. falciparum* parasite burden, a quantitative real-time PCR (qPCR) assay was performed targeting the *msp*2 gene. DNA was extracted from dried blood spots (DBS) on filter paper using a Chelex^®^ 100-based protocol adapted for high-yield amplification. Amplification was performed using genotype-specific probes for the 3D7 and FC27 allelic families, as well as for multiplicity of infection (polyclonal). Quantification was based on a standard curve generated from serial tenfold dilutions ranging from 10⁶ to 10¹ copies/µL of a plasmid containing the *msp*2 gene. Quantification of *P. falciparum* infection was based on a standard curve generated using serial tenfold dilutions of a plasmid containing the *msp*2 gene. A linear regression equation (Ct = -3.32 × log₁₀[copies/µL] + 38.0; R² > 0.98) was used to convert Ct values to parasite densities [9, 10].

### Ethical considerations

The study protocol was reviewed and approved by the Ethics Committee of the Faculty of Medicine at the University of Kindu in the DRC (approval no. UKMED/CEM/2023/014). Written informed consent was obtained from the parents or legal guardians of all children recruited from health centres and participating in the study. For children recruited from schools, parents signed authorisation forms prior to any sampling taking place. Assent was also obtained from children aged seven years and over, in accordance with the Declaration of Helsinki. All procedures ensured confidentiality, voluntary participation, and the right to withdraw at any time.

### Data analysis

Children were classified as symptomatic or asymptomatic based on the presence or absence of fever (axillary temperature > 37.9 °C) and/or malaria-related clinical signs. Parasite density was expressed as the number of *Pfmsp2* gene copies per microliter of blood. Distributions of *msp*2 copy numbers were compared between clinical groups using boxplots. Descriptive statistics including medians, interquartile ranges (IQR), and extreme values were reported for each combination of genotype and clinical status. All data were initially entered into Microsoft Excel 2016 and subsequently analyzed using R software version 3.4.0 (R Foundation for Statistical Computing, Vienna, Austria). Descriptive statistics were used to summarize demographic, clinical, and molecular characteristics of the study population. Comparisons of categorical variables were performed using the chi-squared test or two-proportion test, as appropriate. Continuous variables were compared using the non-parametric Mann–Whitney U test. Logistic regression analyses were conducted to identify factors associated with symptomatic malaria. Crude and adjusted odds ratios (ORs) with 95% confidence intervals (95% CI) were estimated. Logistic regression models were fitted using the stats package. Model discrimination was assessed using the “pROC” package, calibration using the “ResourceSelection” package, and “pseudo-R²” was estimated using the “DescTools” package. Allele frequencies of *Pfmsp2* were calculated as the proportion of each detected allele within its corresponding allelic family (FC27 or 3D7/IC). Multiplicity of infection (MOI) were defined as the detection of more than one *Pfmsp2* allele in a single sample. A two-sided p-value < 0.05 was considered statistically significant.

## Results

### Characteristics of the study population

A total of 522 children were included in the analysis. The mean age differed significantly between symptomatic and asymptomatic groups. Symptomatic children were younger (mean ± SD: 5.77 ± 2.06 years; median: 5.90 years) compared with asymptomatic children (7.25 ± 2.31 years; median: 7.18 years), and this difference was statistically significant (*p* < 0.001). The overall sex distribution showed a higher proportion of males (69.9%) than females (30.1%). Across all study subgroups, male children were more frequently represented than female children. Comparisons within subgroups showed statistically significant differences in sex proportions in most categories, as presented in Table 2.

**Table 2:** Distribution of sex across study subgroups

**Category**	**Female**		Male		Total	*p*-value
	***N***	**%**	*N*	%	*N*	
	**157**	**30.1**	365	69.9	522	
Age group						
0–3 years	32	32.0	68	68.0	100	< 0.001
4–6 years	73	29.2	177	70.8	250	< 0.001
7–12 years	52	30.2	120	69.8	172	< 0.001
Residential area						
Central	81	31.3	178	68.7	259	< 0.001
Peripheral	76	28.9	187	71.1	263	< 0,001
Clinical status						
Symptomatic	84	30.1	195	69.9	279	< 0.001
Asymptomatology	73	30.0	170	70.0	243	< 0.001
Recruitment source						
Sick	115	31.2	253	68.8	368	< 0.001
Prenatal consults	19	47.5	21	52.5	40	0.9
School	23	20.2	91	79.8	114	< 0.001
Malaria infection status						
Negative	85	32.2	179	67.8	264	< 0.001
Positive	72	27.9	186	72.1	258	< 0.001

Data are presented as number (*n*) and percentage (%). *p*-values were obtained using the chi-squared test (or two-proportion test) to compare the proportion of females and males within each subgroup. Statistical significance was defined as *p* < 0.05

### Distribution of children by symptom status according to demographic and malaria infection characteristics

**Fig. 1:**
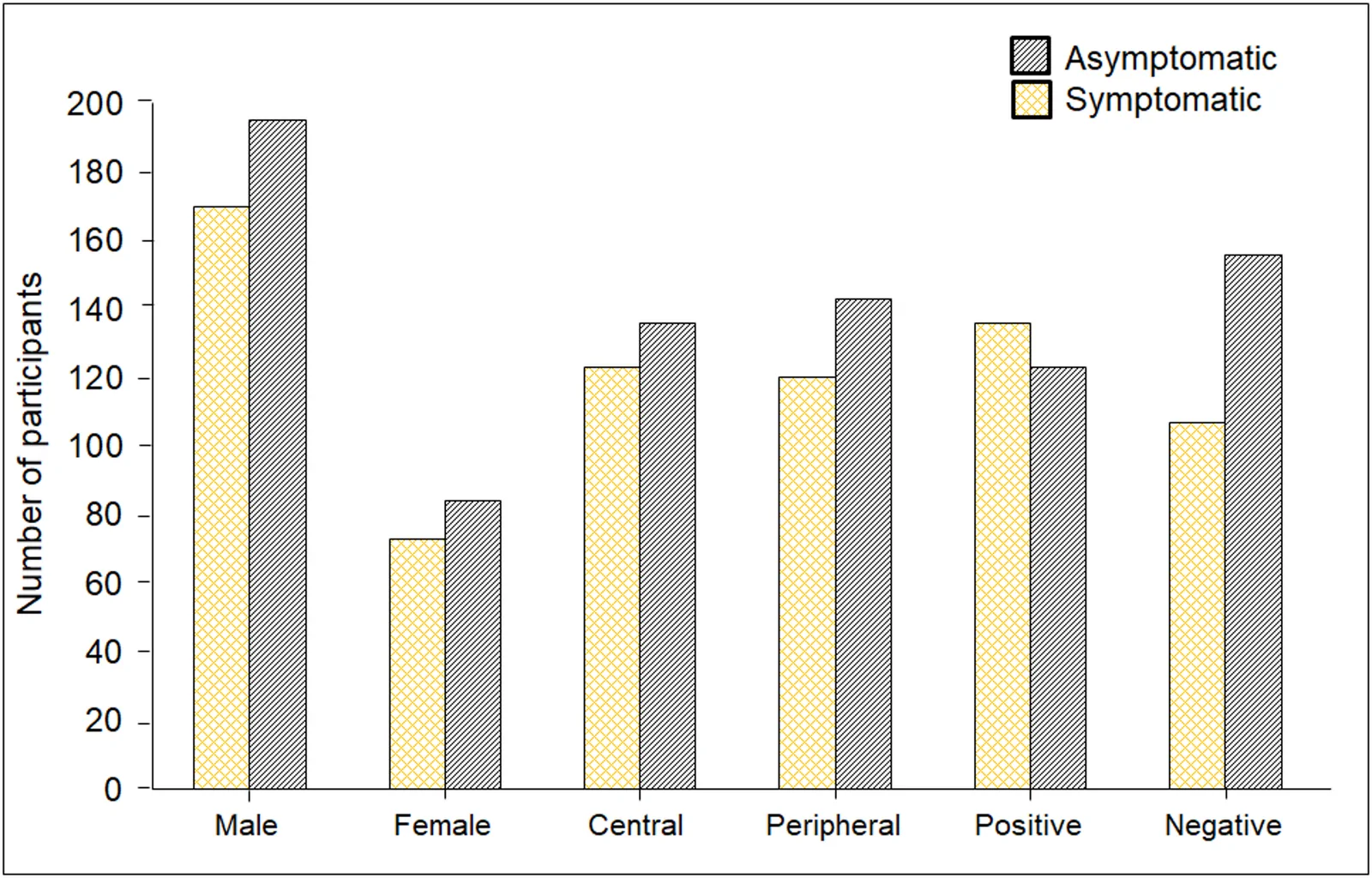
Distribution of children by symptom status according to sex, residential area, and malaria infection status. This figure presents the distribution of children classified as symptomatic or asymptomatic according to key demographic and clinical characteristics, including sex (male and female), residential area (central and peripheral), and malaria infection status (positive and negative). Symptomatic and asymptomatic groups are compared across these variables to illustrate differences in population structure and infection patterns within the study cohort

Figure 1 shows how children are distributed by symptom status across key demographic and clinical variables. A higher proportion of symptomatic cases were observed among males (170/243, 69.9%) and among children from urban areas (123/243, 50.6%), while asymptomatic cases were more frequent among females (84/279, 30.1%) and among those from peripheral areas (143/279, 51.2%). In terms of malaria status, the majority of symptomatic children were infected (136/243, 55.9%), whereas most asymptomatic children tested negative for malaria (156/279, 55.9%). Conversely, among malaria-positive children, symptomatic cases were more frequent (180/258, 69.8%) than asymptomatic cases (78/258, 30.2%). Overall, the figure highlights clear variations in symptom status distribution according to demographic and malaria-related factors, reflecting the heterogeneity of infection presentation in the study population.

### Molecular distribution of *Plasmodium falciparum* infections and *msp2* allelic profiles according to clinical status

**Fig. 2:**
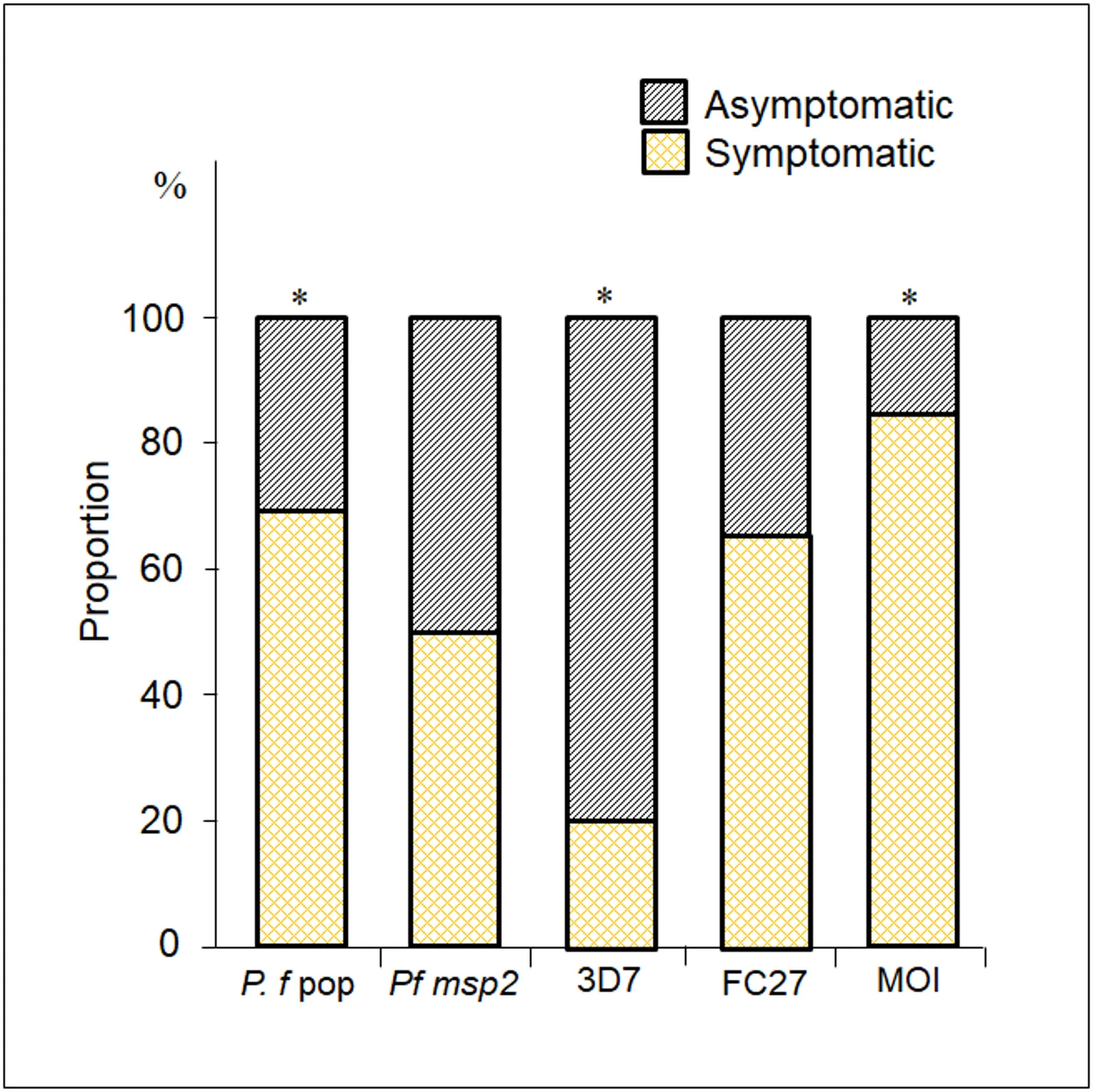
Molecular characterization of *Plasmodium falciparum* infections based on *msp2* allelic families and multiplicity of infection according to clinical status. The figure presents a stepwise molecular characterization of *Plasmodium falciparum* infections in the study population. It begins with all *P. falciparum*-positive samples, followed by those successfully amplified for the *msp2* gene, and subsequently stratifies the distribution of *msp2* allelic families. The analysis includes the main allelic families (3D7 and FC27) as well as multiclonal infections, defined as multiplicity of infection (MOI), corresponding to the presence of both allelic families within the same sample. The distributions are presented according to clinical status (symptomatic and asymptomatic children). Statistical comparisons between groups were performed using the Chi-square test (or Fisher’s exact test where appropriate), with a significance level set at *p* < 0.05, *: significance level set at *p* < 0.05

Among the 258 children infected with *Plasmodium* (49.4% of the total population), *P. falciparum* was identified in 205 cases (79.4%). A significantly higher proportion of these infections occurred in symptomatic children (144/205, 70.2%) (*p* < 0.001). The *msp2* genotyping was performed on all 205 *P. falciparum*-positive samples. Amplification products were obtained in 77 samples (37.5%), which were included in the allelic analysis. Among these 77 samples, the 3D7 allele was the most frequently detected (35/77, 45.4%), followed by FC27 (19/77, 24.6%) and mixed infections (3D7 + FC27) in 23/77 cases (29.8%). The distribution of alleles showed variation according to clinical status. The 3D7 allele was more frequent among asymptomatic children (28/35, 80.0%), whereas FC27 (12/19, 63.2%) and mixed infections (20/23, 86.9%) were more common among symptomatic children (Fig. 2).

### Comparison of *msp2* gene copy number according to clinical status and parasite genotype

**Fig. 3:**
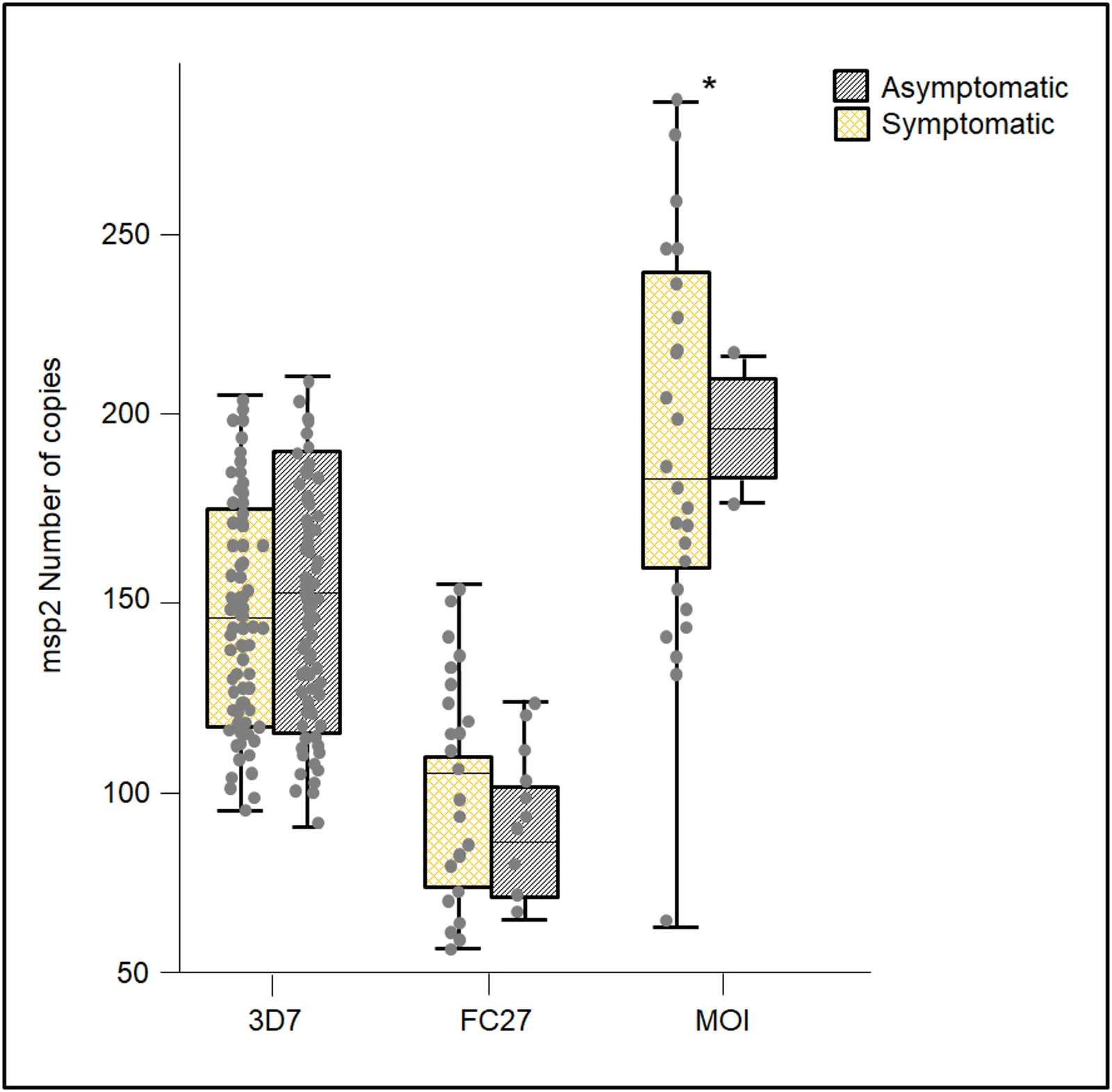
Distribution of *msp2* gene copy number according to clinical status and parasite genotype (3D7, FC27, and mixed infections). Boxplots illustrating the distribution of *msp2* gene copy number in symptomatic and asymptomatic children according to parasite genotype, including 3D7, FC27, and mixed infections (3D7 + FC27). Horizontal lines represent median values, boxes indicate interquartile ranges, and whiskers represent the overall data range. Statistical comparisons between groups were performed using the Mann–Whitney U test, with statistical significance defined as *p* < 0.05, *: significance level set at *p* < 0.05

The distribution of *Pfmsp2* gene copy number among symptomatic and asymptomatic children was examined according to parasite genotype (3D7, FC27, and MOI) (Fig. 3). Among children infected with the 3D7 genotype, symptomatic individuals showed a higher median gene copy number (1.8, IQR: 1.3–2.5) than asymptomatic children (1.1, IQR: 0.8–1.7). For the FC27 genotype, copy numbers were lower in both groups, with medians of 1.3 (IQR: 1.1–1.6) in symptomatic children and 1.0 (IQR: 0.8–1.3) in asymptomatic children. The largest difference was observed in mixed infections, where symptomatic children had a median copy number of 2.9 (IQR: 2.0–4.0), compared with 1.5 (IQR: 1.1–2.1) in asymptomatic carriers. Statistical comparison using the Mann–Whitney U test indicated a significant difference between groups (*p* < 0.001).

### Factors associated with symptomatic malaria: bivariate and multivariable logistic regression analysis

To identify predictors of symptomatic malaria, logistic regression analysis was performed using sociodemographic, parasitological, and molecular variables, including *msp2* genotype and gene copy number (Table 3). In the bivariate analysis, parasitic positivity was strongly associated with symptomatic presentation (OR = 3.84; 95% CI: 2.67–5.53; *p* < 0.001). Regarding parasite genotypes, infections with the FC27 variant showed significantly higher odds of being symptomatic compared with the 3D7 reference group (OR = 6.80; 95% CI: 1.97–23.80; *p* = 0.001). Multiplicity of infections (MOI) were associated with an even greater likelihood of symptomatic malaria (OR = 26.66; 95% CI: 6.13-115.88; *p* < 0.001).

These associations remained significant after multivariable adjustment. Children with FC27 infections had 6.5 fold higher odds of symptomatic malaria compared with those infected with the 3D7 genotype (aOR = 6.50; 95% CI: 1.91–24.55; *p* = 0.004), while mixed infections remained the strongest predictor (aOR = 24.58; 95% CI: 6.10–130.00; *p* = 0.002). Parasitic positivity also remained independently associated with symptomatic malaria (aOR = 3.83; 95% CI: 2.66–5.53; *p* < 0.001). In contrast, *msp2* gene copy number was not significantly associated with clinical status (symptomatic: 1.82 ± 0.37 vs. asymptomatic: 1.71 ± 0.34; aOR = 0.98; 95% CI: 0.97–1.00; *p* = 0.134). No significant associations were observed for age group, sex, or residential area in either crude or adjusted analyses. The final model demonstrated good calibration (Hosmer–Lemeshow *p* = 0.62), acceptable discriminative ability (AUC = 0.79), and moderate explanatory performance (Nagelkerke’s R² = 0.34), indicating that the included predictors explained approximately one-third of the variation in symptomatic status.

**Table 3:** Bivariate and multivariable logistic regression analysis of factors associated with symptomatic malaria

Factor	Category	Number	Symptomatic *n* (%)	Asymptomatic *n* (%)	Crude OR (95% CI)	*p*-value	Adjusted OR (95% CI)	*p*-value
Gender	Female	157	84 (53.5)	73 (46.5)	Reference	-	Reference	-
	Male	365	195 (53.4)	170 (46.6)	1.00 (0.69–1.47)	0.990	0.98 (0.68–1.45)	0.930
Age group	0–3 years	100	55 (55.0)	45 (45.0)	Reference	-	Reference	-
	4–6 years	250	135 (54.0)	115 (46.0)	1.10 (0.66–1.87)	0.660	1.09 (0.70–1.54)	0.690
	7–12 years	172	89 (51.7)	83 (48.3)	0.87 (0.53–1.43)	0.600	0.91 (0.49–1.67)	0.690
Residential area	Center	259	137 (52.9)	122 (47.1)	Reference	-	Reference	-
	Periphery	263	142 (54.0)	121 (46.0)	1.05 (0.74–1.47)	0.800	1.02 (0.73–1.49)	0.870
Malaria status	Negative	264	99 (37.5)	165 (62.5)	Reference	-	Reference	-
	Positive	258	180 (69.8)	78 (30.2)	3.84 (2.67–5.53)	< 0.001	3.83 (2.66–5.53)	< 0.001
*msp2* genotype	3D7	35	7 (20.0)	28 (80.0)	Reference	-	Reference	-
	FC27	19	12 (63.2)	7 (36.8)	6.80 (1.97–23.80)	0.001	6.50 (1.91–24.55)	0.004
	MOI	23	20 (86.9)	3 (13.1)	26.66 (6.13-115.88)	< 0.001	24.58 (6.10–130.00)	0.002
*msp2* gene copy number	Mean ± SD	-	1.82 ± 0.37	1.71 ± 0.34	0.99 (0.98-1.00)	0.258	0.98 (0.97-1.00)	0.134

*Abbreviations: OR *odds ratio, *CI *confidence interval, *MOI *multiplicity of infection, *SD *standard deviation

*p*-values < 0.05 were considered statistically significant. The *msp2* gene copy number was analyzed as a continuous variable

## Discussion

This study offers valuable insights into the genetic diversity and parasite burden of *P. falciparum* among children in Kindu, DRC, a region of stable endemicity. By distinguishing between symptomatic and asymptomatic infections, our findings contribute to understanding the dynamics of parasite-host interactions and the potential role of molecular variants in disease expression.

The study population had an overall infection rate of 49.4%, with a higher prevalence among symptomatic children. However, a significant proportion of asymptomatic children were also found to be carrying Plasmodium. This finding is consistent with reports from other Central African regions where subclinical infections commonly act as a reservoir for the ongoing transmission and maintenance of malaria [10, 13]. Such reservoirs, particularly among school-age children, present a significant challenge to efforts to eliminate the disease, as they perpetuate low-level transmission even when there are no overt clinical symptoms [14, 15].

The predominance of the 3D7 allele (38.0%), followed by multiplicity of infection (34.9%) and FC27 (27.1%) genotypes, is consistent with patterns observed in many other sub-Saharan African countries, though their proportions may vary with ecological context, seasonal transmission intensity, and host immunity. These differences highlight the necessity of local molecular surveillance to capture region-specific patterns [16–19].

Notably, polyclonal infections were significantly more common among symptomatic children, suggesting a possible association rather than a causal relationship between genetic complexity and clinical expression [20]. This supports the hypothesis that co-infections with multiple strains may reflect higher transmission pressure, recent superinfection, or immune system overwhelm, all of which may contribute to infection complexity rather than directly determining pathogenicity [5, 21]. The logistic regression further reinforces this link: children infected with FC27 genotypes were 6.8 times more likely to be symptomatic, while polyclonal infections conferred a 26.6-fold increased odds of clinical malaria. This corroborates earlier evidence associating FC27 with immune evasion and more virulent phenotypes [22].

Although a tendency towards a higher number of *msp*2 genes was observed among asymptomatic cases, particularly those with the 3D7 genotype, symptomatic cases were statistically more prevalent in MOI. This suggests that the genotypic composition of the parasite, rather than parasite load alone, may be a more important factor in determining disease severity, particularly in populations with some immunity [23, 24].

These results highlight the importance of incorporating molecular tools into routine malaria surveillance. As well as monitoring circulating strains, genotyping can contribute to the characterization of infection profiles and may support risk stratification in endemic areas, although its predictive value for clinical severity requires further validation [25, 26]. In the long term, genotype-based monitoring could inform the development of targeted interventions, such as vaccines, and help to prioritise treatment for individuals carrying high-risk alleles [27, 28].

Beyond these potential applications, the interpretation of genotype–clinical associations require careful consideration of the broader genetic and epidemiological context of the study area. In malaria-endemic settings, the clinical expression of infection is influenced not only by parasite genotype, but also by host-related factors such as acquired immunity, age-dependent exposure, and inherited red blood cell traits including hemoglobinopathies or G6PD deficiency [29]. In parallel, parasite population structure, transmission intensity, and local vector ecology may shape the circulation and persistence of specific allelic families such as *msp2* 3D7 and FC27. Therefore, the predominance of certain alleles among symptomatic or asymptomatic children may reflect a complex interaction between host susceptibility, parasite diversity, and environmental transmission dynamics rather than a direct genotype-specific effect alone [30].

Further longitudinal studies are needed to evaluate changes in genotypes and immune responses to specific alleles over time, and the combined impact of these factors on transmission dynamics. This could enhance the predictive accuracy of disease outbreak models and bolster early warning systems in regions with a high disease burden. This study has several limitations, including its cross-sectional design, the limited sample size for *msp2* genotyping (*n* = 77), which may reduce statistical power and limit the robustness of genotype-specific comparisons, and the potential underestimation of low-density asymptomatic infections. This reduced sample size may have influenced the precision of some estimates, leading to wider confidence intervals and reduced statistical power. Therefore, associations between specific *msp2* genotypes and clinical status should be interpreted with caution. In addition, findings from Kindu may not be generalisable to other regions of the DRC. Recruitment biases and unmeasured confounders, such as nutritional status or prior antimalarial treatment, could also have influenced the results. Importantly, key hematological and host-related parameters, including hemoglobin levels, hematocrit, complete blood count (CBC), and hemoglobinopathies, were not available in this study. The absence of these variables limits the ability to fully interpret clinical severity, particularly in relation to anemia and host susceptibility to symptomatic malaria. Nevertheless, our data provide valuable insights into the genetic diversity of *P. falciparum*, highlighting the importance of molecular surveillance and longitudinal studies in improving our understanding of transmission dynamics. In addition, no direct data were available on host genetic traits, local entomological indicators, or parasite population structure, which limits a more comprehensive interpretation of the observed molecular patterns.

## Conclusion

This study highlights the genotypic diversity and parasite burden of *Plasmodium falciparum* in children in Kindu, DRC. Polyclonal infections and the FC27 allele were more frequently associated with symptomatic disease, suggesting that genetic complexity may influence parasite pathogenicity. The results also underscore the critical role of asymptomatic carriers in sustaining malaria transmission. These findings support integrating molecular surveillance tools, such as *msp2* genotyping and qPCR, into routine malaria control strategies, particularly in high-prevalence areas where low-density infections often remain undetected. Incorporating genotypic profiling could enable early detection of virulent strains and guide targeted interventions. Future research should include longitudinal studies and investigation of host immune responses to clarify genotype-specific risks and optimize precision strategies for malaria control and elimination.

## Data Availability

The datasets generated and/or analysed during the current study are not publicly available due to ethical and confidentiality restrictions, but are available from the corresponding author on reasonable request.

## Abbreviations

aOR: Adjusted odds ratio
AUC: Area under the curve
bp: Base pairs
CI: Confidence interval
Ct: Cycle threshold
DBS: Dried blood spots
DNA: Deoxyribonucleic acid
DRC: Democratic Republic of Congo
EDTA: Ethylenediaminetetraacetic acid
FC27: Allelic family FC27 of the Plasmodium falciparum msp2 gene
Giemsa: Giemsa stain
HBsAg: Hepatitis B surface antigen
HIV: Human immunodeficiency virus
IC: Indochina (allelic family 3D7/IC of msp2)
IQR: Interquartile range
MD: Medical doctor
MgCl₂: Magnesium chloride
MOI: Multiplicity of infection
msp2: Merozoite surface protein 2
N: Number
OR: Odds ratio
PCR: Polymerase chain reaction
Pf: Plasmodium falciparum
pfLDH: Plasmodium falciparum lactate dehydrogenase
pLDH: Plasmodium lactate dehydrogenase
qPCR: Quantitative polymerase chain reaction (real-time PCR)
RBC: Red blood cells
RDT: Rapid diagnostic test
rRNA: Ribosomal ribonucleic acid
R²: Coefficient of determination
SD: Standard deviation
SE: Standard error
SSA: Sub-Saharan Africa
UV: Ultraviolet
WHO: World Health Organization
